# Prevalence of human papillomavirus infection in the oropharynx and urine among sexually active men: a comparative study of infection by papillomavirus and other organisms, including *Neisseria gonorrhoeae*, *Chlamydia trachomatis*, *Mycoplasma* spp., and *Ureaplasma* spp

**DOI:** 10.1186/1471-2334-14-43

**Published:** 2014-01-27

**Authors:** Kazufumi Nakashima, Kazuyoshi Shigehara, Shohei Kawaguchi, Akira Wakatsuki, Yoshitomo Kobori, Kazuyoshi Nakashima, Yasunori Ishii, Masayoshi Shimamura, Toshiyuki Sasagawa, Yasuhide Kitagawa, Atsushi Mizokami, Mikio Namiki

**Affiliations:** 1 Department of Integrative Cancer Therapy and Urology, Kanazawa University Graduate School of Medical Science, 13-1, Takaramachi, Kanazawa, Ishikawa 920-8641, Japan; 2 Department of Urology, Ishikawa Prefectural Central Hospital, Kanazawa, Ishikawa, Japan; 3 Wakatsuki Clinic, Osaka City, Osaka, Japan; 4 Department of Urology, Dokkyo Medical School, Koshigaya Hospital, Koshigaya, Saitama, Japan; 5 Nakashima Clinic, Kanazawa, Ishikawa, Japan; 6 Ishii Clinic, Saitama City, Saitama, Japan; 7 Department of Obstetrics & Gynecology, Kanazawa Medical School, Kahoku-gun, Ishikawa, Japan

**Keywords:** Human papillomavirus, Oral infection, Mycoplasma, Ureaplasma, Liquid-based cytology

## Abstract

**Background:**

Oropharyngeal squamous cell carcinoma (OSCC) has shown a gradual increase in male predominance due to the increasing incidence of human papillomavirus (HPV)-associated OSCC. However, the mode of HPV transmission to the oral cavity is poorly understood, and little is known about the epidemiology of oral HPV infection in men. The prevalence rates of HPV, *Neisseria gonorrhoeae*, *Chlamydia trachomatis*, *Mycoplasma* spp., and *Ureaplasma* spp. were compared in the oropharynx (oral cavity) and urine of male Japanese patients attending a sexually transmitted disease clinic.

**Methods:**

The study population consisted of 213 men aged 16 – 70 years old (mean: 34.4 years old). Oropharyngeal gargles and urine were collected, and sedimented cells were preserved in liquid-based cytology solution. After DNA extraction, β-globin and infectious organisms were analyzed by a PCR-based method. The HPV genotype was determined by HPV GenoArray test.

**Results:**

β-Globin was positive in 100% and 97.7% of oral and urine samples, respectively. HPV detection rates were 18.8% and 22.1% in oral and urine samples, respectively, suggesting that the prevalence of HPV infection in the oral cavity was similar to that in the urinary tract. *N. gonorrhoeae* was more prevalent in oral (15.6%) than urine samples (9.1%), whereas *C. trachomatis* was detected more frequently in urine (15.9%) than oral samples (4.2%). The detection rates of *M. genitalium*, *M. hominis*, and *Ureaplasma* spp. were 5.2%, 10.3%, and 16.0% in oral samples, and 7.7%, 6.3%, and 19.2% in urine, respectively. There were no significant differences in the detection rates of *Mycoplasma* spp. and *Ureaplasma* spp. between anatomical locations. The distribution of HPV types were similar in oral and urine samples, and HPV16 was the most common type. The majority of men with HPV infection in both the oral cavity and urine had concordant oral and urinary HPV infection. The presence of urinary HPV infection was an independent risk factor of oral HPV infection, with an odds ratio of 3.39 (95% CI: 1.49 – 7.71), whereas oral gonococcal infection was inversely correlated with oral HPV infection (odds ratio: 0.096; 95% CI: 0.01 – 0.77).

**Conclusions:**

Oral HPV infection commonly occurs in sexually active men, and is significantly correlated with urinary HPV infection.

## Background

Human papillomavirus (HPV) infection is a necessary element for the development of virtually all cases of cervical cancer in women [1], and has also been identified as a risk factor for the development of anal, penile, and oropharyngeal cancers [2-4]. In particular, oropharyngeal squamous cell carcinoma (OSCC) has shown a gradual increase in male predominance since the 1970s, despite reductions in smoking, as a result of the increasing incidence of HPV-associated OSCC. Indeed, a recent study indicated that HPV detection represented about 70% of all OSCCs in the USA in 2007, compared with 10% in the 1980s [5], and another large-scale study indicated that the HPV prevalence of 323 OSCC cases was 63.8% (206 cases) [6]. A Japanese epidemiological report in 2008 by the Ministry of Health, Labour and Welfare found that the incidence of OSCC has also been gradually increasing over the last three decades in Japan, which is likely due to the increase in HPV-positive cases [7]. HPV-associated OSCC is now a well-defined entity with well-known characteristics that include young age, good performance status, male gender, nonsmoking, and high-risk sexual behavior [6,8].

Cervical HPV infection appears to occur frequently within a few years of sexual debut, and its prevalence decreases with increasing age from the peak prevalence in younger women [9]. Sexually transmitted cervical HPV infection, when it persists for 10 – 15 years, presents a risk of developing precancerous lesions of the cervix, which can progress to invasive cervical cancer. On the other hand, HPV-associated OSCC is also thought to be induced by oropharyngeal HPV infection. However, the mode of transmission of HPV to the oral cavity is less well understood, and little is known about the epidemiology of oral pharyngeal HPV infection in men.

In the present study, we investigated the prevalence of HPV infection and HPV types in the oropharynx (oral cavity) and urine of male Japanese patients who attended a sexually transmitted disease (STD) outpatient clinic, and the concordance of infected HPV types between these two infection sites. In addition, the infection statuses of *Neisseria gonorrhoeae*, *Chlamydia trachomatis*, *Mycoplasma* spp., and *Ureaplasma* spp. were also examined in oral and urine samples, and we investigated the possible association of oral HPV infection with these infectious organisms.

## Methods

### Study subjects

A total of 213 male Japanese patients who attended the STD outpatient clinic of Kanazawa University Hospital (Kanazawa, Japan), Ishikawa Prefectural Central Hospital (Kanazawa, Japan), Dokkyo Medical School, Koshigaya Hospital (Koshigaya, Japan), Wakatsuki Clinic (Osaka, Japan), and Nakashima Clinic (Kanazawa, Japan) were enrolled in this study. All participants were men who had sex with women, and homosexual or bisexual men were excluded from the study. The ethics committee of Kanazawa University Graduate School of Medicine approved this study. Oropharyngeal gargle and urine samples were obtained from each subject after obtaining their written informed consent. A clinical diagnosis of urethritis was defined as ≥  5 polymorphonuclear leukocytes per high-power field in collected urethral swabs.

To obtain oral specimens, each subject swished 15 mL of normal saline around their oral cavity, gargled with their head tilted back for 10 – 20 s, and then expectorated into a specimen cup. In addition, all urine from one urination for each patient was collected in an individual urine cup, and 15 mL was placed into a separate tube. Each sample (15 mL) was centrifuged at approximately 1500  ×  *g* for 10 min, and the sediment was placed into a separate tube containing 2.5 mL of preservative solution for liquid-based cytology (LiquiPrep; LGM International Inc., Melbourne, FL, USA) and stored at 4°C until use.

### HPV-DNA test and genotyping

Aliquots of 800 μL of preservative solution containing cell samples were centrifuged at approximately 1500  ×  *g* for 10 min, and the supernatants were discarded. The cell pellets were washed twice with 300 μL of 10 mmol/L Tris-HCl (pH 8.0). DNA was extracted from the cells using a DNA extraction kit (SMI Test; G&G Science Co., Fukushima, Japan) according to the manufacturer’s instructions. The β-globin gene was first amplified to confirm the adequacy of the extracted DNA in all samples. In β-globin-positive samples, HPV-DNA was tested by modified GP5+/GP6+ polymerase chain reaction (PCR) as described previously [10].

HPV genotyping was performed using HPV GenoArray Test Kits (HybriBio Limited, Chaozhou, China) according to the manufacturer’s protocol. This assay can be used to determine 21 HPV genotypes, consisting of 14 high-risk HPV types (16, 18, 31, 33, 35, 39, 45, 51, 52, 56, 58, 59, 66, and 68), 5 low-risk HPV types (6, 11, 42, 43, and 44), and two unknown-risk types (53 and CP8304), by flow-through hybridization using HPV-DNA amplified by PCR [11]. Internal control and HPV genotype were visualized simultaneously as spots at the position of control and each HPV type probe on the same membranes [11]. This is a kit that has recently become commercially available, and has shown good agreement (93.8%) in detection of HPV types with the results of the Amplicor HPV test, as described previously [12].

### Detection of *N. gonorrhoeae*, *C. trachomatis*, *M. genitalium*, *M. hominis*, and *Ureaplasma* spp. (biovars 1 and 2)

*N. gonorrhoeae* was detected in each liquid-based sample by PCR using specific primers targeting the *N. gonorrhoeae orf1* gene [13]. For the 427 urethral swabs, the sensitivity, specificity, positive predictive value, negative predictive value for this PCR method were reported to be 100%, 98%, 99.7%, and 100%, respectively. The primer for *C. trachomatis* amplified a 205-bp fragment of the 16S rRNA gene, as described previously, and had sensitivity and specificity of 97.9% and 98.7%, respectively [14].

*Mycoplasma genitalium*, *Mycoplasma hominis*, and *Ureaplasma* spp. (biovars 1 and 2) were detected by multiplex PCR assay as described by Stellrecht *et al*. [15,16]. This Multiplex PCR is a simple and convenient method performed with primers specific for highly conserved regions in the urease gene of *Ureaplasma* spp., the 140-kDa adhesion protein gene of *M. genitalium*, and the 16S rRNA gene of *M. hominis*, yielding bands of 403 – 448 bp, 282 bp, and 334 bp, respectively [16]. The overall sensitivity, specificity, and positive and negative predictive values of multiplex PCR analyses for detection of *Mycoplasma* spp. and *Ureaplasma* spp. were 87%, 96%, 94%, and 93%, respectively, with an analytical lower limit of detection of <  12.5 colony forming units (cfu) for *Ureaplasma* spp., *M. genitalium*, and *M. hominis*, even if these organisms were mixed together.

### Statistical analysis

The chi-square test was used to compare HPV-positive rates between the two anatomical sites among all 213 patients. Univariate and multivariate analysis using unconditional direct logistic regression analysis for all variables was performed to determine the risk factors for detection of oral HPV infection, and the odds ratios (OR) and 95% confidence intervals (CI) were calculated. The SPSS statistical software package (version 17.0; SPSS Inc., Chicago, IL) was used for all analyses, and *P*  <  0.05 was taken to indicate statistical significance.

## Results

Among the 213 participants aged 16 – 70 years (mean ± SD; 34.3  ±  10.2 years), 91 (43%) had urethritis, comprised of 17 gonococcal, 31 chlamydial, 2 gonococcal and chlamydial, and 41 non-gonococcal and non-chlamydial infections (Table 1). The other 122 patients had no evidence of urethritis. Among these 122 patients, two with painful clusters of genital sores consisting of vesicles on the penile surface were diagnosed as having *Herpes genitalis*, and *Trichomonas* was incidentally identified in one patient based on urinary microscopic examination. There were no patients with any pharyngitis-like symptoms.

**Table 1:** **Patient backgrounds (*n***  **=  213)**

**Variables**	***n***
Mean age (range)	34.3 (16 – 70)
Number of patients with urethritis	91 (43%)
Gonococcal	17
Chlamydial	31
Gonococcal and chlamydial	2
Non-gonococcal and non-chlamydial	41
Number of patients non-urethritis	122 (57%)
*Herpes genitalis*	2
Asymptomatic *Trichomonas* infection	1
Asymptomatic (for STD check)	119

STD, sexually transmitted disease.

Positive amplification of the β-globin gene was observed in all 213 (100%) of the oral samples and in 208 (97.7%) of the urine samples (Table 2). Among the β-globin-positive samples, the HPV detection rates were 18.8% in oral samples and 22.1% in urine samples. High-risk HPV was detected in 14.1% of oral samples and in 15.4% of urine samples. There were no significant differences in the prevalence raters of HPV or high-risk HPV between oral and urine samples. *N. gonorrhoeae* was more prevalent in oral samples (15.6%) than in urine samples (9.1%), whereas *C. trachomatis* was detected more frequently in urine samples (15.9%) than in those from the oral cavity (4.2%). On the other hand, the detection rates of *M. genitalium*, *M. hominis*, and *Ureaplasma* spp. were 5.2%, 10.3%, and 16.0% in the oral cavity and 7.7%, 6.3%, and 19.2% in urine, respectively. The differences in detection rates between oral and urine samples were not significant.

**Table 2:** Results of PCR analysis of oral samples and urine samples by liquid-based cytology

	**Oral cavity**	**Urine**	***P*-value**
	**(*n*** **= 213)**	**(*n*** **= 213)**	
Number of adequate samples (%)	213 (100%)	208 (97.7%)	
HPV detection rate (%)	40 (18.8%)	46 (22.1%)	0.331
High-risk HPV detection rate (%)	30 (14.1%)	32 (15.4%)	0.575
Low-risk HPV detection rate (%)	12 (5.6%)	19 (9.1%)	0.276
Frequency of single-type HPV infection (%)	30 (75.0%)	24 (52.2%)	0.029
*N. gonorrhoeae* (%)	36 (15.6%)	19 (9.1%)	0.018
*C. trachomatis* (%)	9 (4.2%)	33 (15.9%)	< 0.001
*M. genitalium* (%)	11 (5.2%)	16 (7.7%)	0.290
*M. hominis* (%)	22 (10.3%)	13 (6.3%)	0.130
*Ureaplasma* spp. (%)	34 (16.0%)	40 (19.2%)	0.378

PCR, polymerase chain reaction; HPV, human papillomavirus.

The HPV type distributions were similar in oral and urinary samples, and HPV16 was the most common type, followed by types 6, 18, 11, and 33 (Table 3). However, single-type HPV infection was frequently identified in the oral cavity (30/40 cases; 75.0%), whereas multiple-type HPV infection was found more frequently in urine samples than in oral samples (Table 2). Moreover, there were 18 cases with HPV infection at both anatomical sites (Table 4). Among these cases, the detected HPV types in oral and urinary samples were in complete agreement in 8 cases (44.4%), and were consistently similar in 16 cases (88.9%).

**Table 3:** Type-specific prevalence rates of HPV in oral and urine samples

**HPV type**	**Oral cavity (*n*** **= 40)**	**Urine (*n*** **= 46)**
	***n *** **(%)**	***n *** **(%)**
**High-risk type**		
16	17 (42.5)	19 (41.3)
18	7 (17.5)	11 (23.9)
33	5 (12.5)	6 (13.0)
39	-	1 (2.2)
52	1 (2.5)	3 (6.5)
53	-	2 (4.3)
58	1 (2.5)	3 (6.5)
59	1 (2.5)	2 (4.3)
66	-	1 (2.2)
**Low-risk type**		
6	8 (20.0)	11 (23.9)
11	5 (12.5)	10 (21.7)
**Unknown type**		
	1 (2.5)	2 (4.3)

HPV, human papillomavirus.

**Table 4:** Details of HPV types in cases in which HPV was detected from both oral and urine samples

	**Oral cavity**	**Urine**
Case 1	16	16, 52
Case 2	16	16, 18
Case 3	16, 33	11, 16, 33
Case 4	16, 33	33
Case 5	16, 33	16, 33
Case 6	18	18
Case 7	18	18
Case 8	6	18
Case 9	6, 16	6
Case 10	16	16, 53
Case 11	16	6, 16
Case 12	6	6
Case 13	16	16
Case 14	16	16
Case 15	16	16
Case 16	6, 11	11
Case 17	18	11
Case 18	18	18

HPV was detected in both oral and urine samples from 16 cases.

HPV, human papillomavirus.

In addition, we attempted to identify the risk factors for oral HPV infection. Multivariate analysis indicated that the presence of urinary HPV infection was an independent risk factor for oral HPV infection, with an odds ratio of 3.39 (95% CI: 1.49 – 7.71) (Table 5). Moreover, oral gonococcal infection was inversely correlated with oral HPV infection (odds ratio: 0.096; 95% CI: 0.01 – 0.77). On the other hand, age, presence of urethritis, and coinfection of the urinary tract with *C. trachomatis*, *M. genitalium*, *M. hominis*, *Ureaplasma* spp., and *N. gonorrhoeae* were not correlated with oral HPV infection.

**Table 5:** Univariate and multivariate analyses of risk factors for detection of any HPV infection in oral samples

	***n***	**Oral HPV infection**		**Univariate analysis**			**Multivariate analysis**		
**Variables**		**Positive**	**Negative**	**Odds ratio**	**95% CI**	***P***	**Odds ratio**	**95% CI**	***P***
**Age (yo)**									
< 35	115	23	92	1.0 (referent)			1.0 (referent)		
≥ 35	98	17	81	0.65	0.12–1.70	0.647	0.71	0.32–1.58	0.402
**Urine HPV infection**									
negative	167	22	145	1.0 (referent)			1.0 (referent)		
positive	46	18	28	4.24	2.02–8.91	<0.001	3.39	1.49–7.71	0.004
**Urethritis**									
negative	122	28	94	1.0 (referent)			1.0 (referent)		
positive	91	12	79	0.51	0.24–1.07	0.0711	0.78	0.32–1.81	0.532
**Oral samples**									
***N. gonorrhoeae***: negative	177	38	139	1.0 (referent)			1.0 (referent)		
positive	36	2	34	0.22	0.05–0.94	0.016	0.096	0.01–0.77	0.027
***C. trachomatis***: negative	204	39	165	1.0 (referent)			1.0 (referent)		
positive	9	1	8	0.53	0.06–4.35	0.470	0.39	0.03–4.69	0.460
***M. genitalium***: negative	202	39	163	1.0 (referent)			1.0 (referent)		
positive	11	1	10	0.42	0.05–3.36	0.353	0.28	0.02–3.10	0.296
***M. hominis***: negative	191	35	156	1.0 (referent)			1.0 (referent)		
positive	22	5	17	1.31	0.45–3.79	0.398	0.68	0.10–4.50	0.692
***Ureaplasma***: negative	179	33	146	1.0 (referent)			1.0 (referent)		
positive	34	7	27	1.15	0.46–2.86	0.464	0.52	0.09–3.16	0.478
**Any organisms**: negative	126	26	100	1.0 (referent)			1.0 (referent)		
positive	87	14	73	0.74	0.36–1.51	0.405	3.84	0.59–27.92	0.184
**Urine samples**									
***N. gonorrhoeae***: negative	194	38	156	1.0 (referent)			1.0 (referent)		
positive	19	2	17	0.48	0.11–2.18	0.267	0.56	0.04–7.62	0.663
***C. trachomatis***: negative	180	38	142	1.0 (referent)			1.0 (referent)		
positive	33	2	31	0.24	0.06–1.05	0.051	0.21	0.02–2.10	0.186
***M. genitalium***: negative	197	39	158	1.0 (referent)			1.0 (referent)		
positive	16	1	15	0.27	0.04–2.11	0.158	0.64	0.05–8.77	0.737
***M. hominis***: negative	200	38	162	1.0 (referent)			1.0 (referent)		
positive	13	2	11	0.78	0.17–3.64	0.545	0.42	0.06–2.96	0.380
***Ureaplasma***: negative	173	31	142	1.0 (referent)			1.0 (referent)		
positive	40	9	31	1.33	0.58–3.07	0.320	1.29	0.12–10.60	0.814
**Any organisms**: negative	116	26	90	1.0 (referent)			1.0 (referent)		
positive	97	14	83	0.58	029–1.19	0.140	0.94	0.09–9.43	0.958

HPV, human papillomavirus.

## Discussion

There is substantial molecular evidence suggesting a role for HPV infection in the pathogenesis of OSCC, and the incidence of HPV-associated OSCC has been gradually increasing worldwide [5,6]. The development of oral HPV infection is essential in the first phase of the development of HPV-associated OSCC, and oral sexual intercourse is suspected to be a route of oral HPV infection. Indeed, a case-control study of HPV and OSCC demonstrated that oral HPV infection is strongly associated with OSCC, and indicated that a high lifetime number of oral sex partners (more than 6 partners) was an independent risk factor for OSCC, with an odds ratio of 3.4 (95% CI: 1.3 – 8.8) [17]. However, little information is available regarding the epidemiological status of oral HPV infection, especially in men. Thus, the prevalence of HPV infection in the oropharynx (oral cavity) and urine in men was examined in the present study. We found that the prevalence rates of HPV infection among Japanese men who attended an STD clinic were 18.8% and 22.1% in oral and urine samples, respectively. This is the first study to compare the HPV detection rate of the oral cavity and urine, and the HPV detection rate in the oral cavity was similar to that in urine among these subjects.

D'Souza *et al.* investigated the prevalence of oropharyngeal HPV infection using mouthwash among 332 control patients who attended an outpatient otolaryngology clinic and 210 college-age men in the USA [18]. HPV infection was detected in 4.8% of 332 control patients from the outpatient clinic and in 2.9% of 210 college-age men, and the increased lifetime numbers of oral sex partners or open-mouth kissing partners were found to be risk factors for oral HPV infection. Kreimer *et al.* detected oral HPV infection in 4.0% (67 cases) of 1608 healthy men aged 18 – 78 from the USA, Mexico, and Brazil using the Roche linear array HPV genotyping assay [19]. In addition, a recent systematic review indicated that 1.3% of 3977 healthy subjects had oral HPV16, 3.5% of 4441 subjects had high-risk HPV, and 4.5% of 4070 subjects were positive for any HPV. Men and women had similar prevalence rates of any oral HPV (4.6% and 4.4%, respectively) [20]. These previous reports suggest that the oropharyngeal HPV prevalence rate among healthy subjects is approximately 3% – 4%, and that HPV is rarely detected in oral specimens compared with genital or urine samples from healthy individuals.

However, the prevalence in our subjects (18.8%) was much higher compared with those reported in previous studies, which was likely due to the difference in study populations. Our study was performed in male patients who attended an STD clinic, and therefore they represented a population that would have risk factors for sexually transmitted infection. Indeed, the prevalence of oral HPV infection in women was also reported to differ according to the study population, and its prevalence was higher (23.6%) in women who attended STD outpatient clinics [21] compared with healthy women [22,23]. In addition, a cohort study of 212 men aged 18 – 24 indicated that nearly 20% of sexually active male university students had evidence of oral HPV infection within 12 months [24]. Moreover, the HPV prevalence rate in oral samples among our subjects was approximately equivalent to that in urine samples, which is a common HPV detection site in sexual active men [11,25]. Oral HPV infection may also be a sexually transmitted infection similar to genital HPV infection. However, in the present study there was a lack of data regarding the ways and frequencies of sexual contact or number of sexual partners in our population. Further epidemiological studies including such sexual information and healthy controls are required to reach more definitive conclusions.

We found that type-specific HPV prevalence was similar in oral and urinary samples, and HPV16 was the most common type identified in the present study. In addition, the majority of men with HPV infection in both the oral cavity and urine specimens had concordant oral and urinary HPV infection, suggesting that oral HPV infection in men may also occur through oral and genital contact with women. Indeed, the present study demonstrated that the presence of urinary HPV infection is an independent risk factor for oral HPV infection, with an odds ratio of 3.39.

Moreover, single-type HPV infection was frequently identified in the oral cavity, and single HPV16 was the most common type. Many epidemiologic studies indicated that HPV16 is the most common HPV type detected from HPV-associated OSCC [17,18], which is consistent with our findings. Multiple-type HPV infection is commonly observed in the cervix of women, low-grade cervical intraneoplasia (CIN), and male external genitalia. On the other hand, single-type HPV infection is an indicator of high-grade CIN or cervical cancer [26]. Although this difference between oral and urinary HPV infection has not been clarified, the oral cavity in men may be easily infected by HPV16. Alternatively, HPV16 infection may be persistent in the oral cavity in men. However, further studies of the natural history of oral HPV infection in men are required to confirm this hypothesis.

We performed another additional study to investigate the possible associations between oral HPV infection and other organisms responsible for sexually transmitted infections (STIs). In a previous study, we investigated the prevalence rates of *Mycoplasma* spp. and *Ureaplasma* spp. using liquid-based cytology samples of urine [25]. *M. genitalium*, *M. hominis*, *Ureaplasma parvum*, and *Ureaplasma urealyticum* were detected in 14.5%, 10.9%, 6.5%, and 12.3% of men with urethritis, whereas these species were detected in 3.3%, 2.0%, 4.7%, and 2.7% of controls, respectively [25]. Takahashi *et al.* also reported prevalence rates of 1%, 4%, 12%, and 23% for *M. genitalium*, *M. hominis*, *U. parvum*, and *U. urealyticum*, respectively, in urine samples from 100 asymptomatic healthy Japanese men [27]. These findings are different from the present results. In particular, *M. genitalium* has been widely accepted as an agent of STIs, and the differences between these studies may have been due to differences in the sample size and characteristics of the study populations as well as the detection methods used. On the other hand, little information is available regarding the prevalence rates of *Mycoplasma* spp. and *Ureaplasma* spp. in the male oral cavity. Sackel *et al.* reported that *M. hominis* and *Ureaplasma* spp. were recovered from pharyngeal samples obtained from 149 (14.3%) and 154 (14.8%), respectively, of 1044 men and women who attended clinics in Boston, USA, which were equivalent to the findings of the present study [28]. There were no significant differences in the detection rates of *M. genitalium*, *M. hominis*, and *Ureaplasma* spp. in males between the oral cavity and urine samples, suggesting that genital *Mycoplasma* spp. and *Ureaplasma* spp. could frequently infect the male oral cavity.

It has been reported that the presence of infection in the urinary tract is an independent risk factor for HPV infection [15]. HPV generally infects the basal layer of the mucosa, and inflammation and microinjury of the urothelium caused by *N. gonorrhoeae*, *C. trachomatis*, and/or *M. genitalium* are likely to facilitate access of HPV to the basal cells of the urothelium in the urethra. However, multivariate analysis indicated that oral gonococcal infection was a negative factor for oral HPV infection in the present study. One possible reason or this observation is that there were no patients with pharyngitis-like symptoms. Most oropharyngeal gonococcal or chlamydial infections are asymptomatic without inflammation of the oropharynx [29], and it may not be possible to demonstrate that other isolated microorganisms, such as *N. gonorrhoeae*, *C. trachomatis*, and *M. genitalium*, were risk factors for the detection of oral HPV infection. Alternatively, the population size in the present study was small, and further large-scale studies will be required to reach more definitive conclusions.

In the present study, a liquid-based cytological procedure, which is commonly used in cervical cancer screening, was used for detection of HPV in oropharyngeal gargle and urine samples. Urine is generally considered unsuitable for the detection of HPV due to the poor β-globin detection rate (< 50%) [30]. However, our previous study indicated that the liquid-based cytological procedure is useful for HPV detection in urine samples from men, and we found that the detection rate of the β-globin gene was >  97% in urine from healthy men and male patients with urethritis [25]. We found an excellent detection rate (100%) of β-globin in oropharyngeal gargle samples using a liquid-based cytological procedure, suggesting that this method, which is recommended for testing oropharyngeal STIs, such as *N. gonorrhoeae*, *C. trachomatis*, *Mycoplasma* spp., and *Ureaplasma* spp., is also suitable for detection of HPV in oropharyngeal gargle samples.

## Conclusion

In conclusion, oral HPV infection is common in sexually active men, and its prevalence rate was equivalent to that in urine samples. In addition, oral HPV infection was significantly correlated with urinary HPV infection.

## Abbreviations

HPV: Human papillomavirus; OSCC: Oropharyngeal squamous cell carcinoma; STD: Sexually transmitted disease; PCR: Polymerase chain reaction; CIN: Cervical intraneoplasia; STI: Sexually transmitted infection.

## Competing interests

The authors declare they have no conflicts of interest.

## Authors’ contributions

All authors made substantial contributions to conception and design, or acquisition of data. KN, KS, and SK were in charge of the analysis and interpretation of all data. KN drafted major portions of the initial manuscript, and KS, TS, and MN helped in writing the final manuscript. All authors have read and approved the final manuscript.

## Pre-publication history

The pre-publication history for this paper can be accessed here:

http://www.biomedcentral.com/1471-2334/14/43/prepub
